# Single Nucleotide Polymorphisms in the Vitamin D Receptor Gene (*VDR*) May Have an Impact on Acute Pancreatitis (AP) Development: A Prospective Study in Populations of AP Patients and Alcohol-Abuse Controls

**DOI:** 10.3390/ijms19071919

**Published:** 2018-06-29

**Authors:** Anna Cieślińska, Elżbieta Kostyra, Ewa Fiedorowicz, Jadwiga Snarska, Natalia Kordulewska, Krzysztof Kiper, Huub F. J. Savelkoul

**Affiliations:** 1 Faculty of Biology and Biotechnology, University of Warmia and Mazury, 10-719 Olsztyn, Poland; anna.cieslinska@uwm.edu.pl (A.C.); elzbieta.kostyra@uwm.edu.pl (E.K.); ewa.kuzbida@uwm.edu.pl (E.F.); natalia.smulska@uwm.edu.pl (N.K.); 2 Department of General Surgery, Faculty of Medical Sciences, University of Warmia and Mazury, 10-719 Olsztyn, Poland; jct4444@onet.pl; 3 Faculty of Medicine, Rzeszów University, 35-310 Rzeszów, Poland; krzychukiper@gmail.com; 4 Cell Biology and Immunology Group, Wageningen University, 6700 AG Wageningen, The Netherlands

**Keywords:** acute pancreatitis, polymorphism, vitamin D receptor, vitamin D, SNP analysis

## Abstract

Vitamin D imbalance is suggested to be associated with the development of pancreatitis. Single nucleotide polymorphisms (SNPs), Apa-1, Bsm-1, Fok-1, and Taq-1, in the vitamin D receptor gene (*VDR*) are known in various diseases, but not yet in pancreatitis. The aim of this study was to explore possible associations of the four SNPs in the *VDR* receptor gene in a population of acute pancreatitis patients and alcohol-abuse controls, and to investigate the association with acute pancreatitis (AP) susceptibility. The study population (*n* = 239) included acute pancreatitis patients (*n* = 129) and an alcohol-abuse control group (*n* = 110). All patients met the Diagnostic and Statistical Manual of Mental Disorders (DSM IV) criteria for alcohol dependence. DNA was extracted from peripheral leukocytes and analyzed for *VDR* polymorphisms using a polymerase chain reaction-restriction fragment length polymorphism (PCR-RFLP) method. Odd ratios (ORs) and 95% confidence intervals (CIs) were calculated using logistic regression analysis. To date, we have found allele T in Taq-1 (OR = 2.61; 95% CI: 1.68–4.03; *p* < 0.0001) to be almost three times more frequent in the AP group compared to the alcohol-abuse control patients. Polymorphism Taq-1 occurring in the vitamin D receptor may have an impact on the development of acute pancreatitis due to the lack of the protective role of vitamin D.

## 1. Introduction

Acute pancreatitis (AP) is a multifactorial disease that develops due to pancreatic ischaemia, pancreatic bile duct obstruction, or activation of pancreatic protease and production of pro-inflammatory cytokines [1,2]. It has an unpredictable course and the development of prognostic symptoms can determine patients at high risk of a severe course of this illness who require appropriate treatment and intensive care [3,4]. AP severity is related to demographic factors (age and obesity), local complications (pancreatic necrosis and fluid collection), and organ failure, as well as genetic factors [5]. Nevertheless, gallstones (cholelithiasis) and alcohol abuse are the main risk factors for acute pancreatitis.

Since vitamin D receptors have been found in several human brain structures [6,7,8], its role has become more important in various psychiatric issues. Low vitamin D levels have been associated with schizophrenia, depression, pancreatic cancer, and autism [8,9,10]. Vitamin D levels are also usually reduced in alcohol-abuse patients [11,12]. Lieber et al. [13] correlated low vitamin D serum levels in chronic alcoholics with pancreatic insufficiency. According to the research of Kim et al. [14], concentrations of the inactive form of vitamin D, 25-hydroxyvitamin D (25(OH)D_3_), were significantly lower in dogs with AP in comparison to healthy dogs. The active vitamin D metabolite, 1,25-dihydroxyvitamin D (1,25-dihydroxycholecalciferol or 1,25(OH)_2_D_3_), selectively binds to a specific vitamin D receptor (*VDR*) [15]. This *VDR* subsequently regulates transcription of genes that are involved in calcium metabolism, cellular proliferation and differentiation, aging, and T-cell mediated immune responses [16,17,18,19,20,21,22,23].

*VDR* are also expressed on pancreatic β cells [24], and may play an essential role in maintaining normal insulin levels in accordance to glucose concentrations and to maintain glucose tolerance [25,26,27]. Because vitamin D acts through *VDR*, their impairment or reduced functionality, e.g., as a result of polymorphisms occurring in the *VDR* gene, may have a crucial impact on the balance in the vitamin D concentration in the circulation, and the final metabolite activity throughout the body.

According to Valdivielso and Fernandez [19], RFLP (Restriction Fragments Length Polymorphism) assays were used to identify four important polymorphisms in the *VDR* gene. Polymorphism detected in intron 8 by the restriction enzyme, Apa-1 (rs7975232), results in T (variant “A”) changing into G (variant “a”) [28]. Bsm-1 polymorphism (rs1544410), also located in intron 8, causes a change of A (variant “b”) into G (variant “B”) [29]. Taq-1 (rs731236) polymorphism (T as variant “T” changed into C as “t” variant) observed in exon 9 leads to a silent mutation in codon 352 [30]. The missense Fok-1 (rs2228570) transition, located in exon 2, results in *VDR* protein variants of 427 amino acids (f (T)) and of 424 amino acids (F (C)). In some cell types, the latter one (F variant) results in a more active form of the protein [31,32,33,34].

The aim of this study was to investigate the genetic association of the four different *VDR* polymorphisms (Apa-1, Bsm-1, Fok-1, Taq-1) with susceptibility to the development of acute pancreatitis compared to a control group of alcohol-abuse patients.

## 2. Results

The observed genotype frequencies of Apa-1 (rs7975232), Bsm-1 (rs1544410), Fok-1 (rs2228570), and Taq-1 (rs731236) polymorphisms in the *VDR* gene were studied in 110 alcohol-abuse controls with diagnosed alcohol-abuse and 129 patients with acute pancreatitis. The data obtained conformed to the Hardy-Weinberg equilibrium. In the whole study population, three genotypes at the *VDR* gene polymorphic site, Apa-1 (rs7975232), were identified: AA, Aa, and aa, with a number of genotypes of 81, 104, and 54, respectively.

At the Bsm-1 (rs1544410) *VDR* gene polymorphic site the frequency of the alleles, B and b, were determined in our study’s alcohol-abuse controls and in those diagnosed with AP. Of the total 239 participants, three genotypes (BB, Bb, bb) were identified: 28 had genotype BB, 124 had Bb, and 87 had bb.

At the *VDR* gene polymorphic site, Fok-1 (rs2228570); the three FF, Ff, and ff genotypes were identified. Of the total 239 participants, 90 had genotype FF, 104 had genotype *Ff*, and 45 had genotype *ff*.

At the *VDR* gene polymorphic site, Taq-1 (rs731236); the three TT, Tt, and tt genotypes were identified, with a 0.61 T-allele frequency in the entire research population (control and AP groups). Of the total 239 participants, 83 carried the TT genotype, 127 carried the *Tt* genotype, and 29 carried the *tt* genotype.

These results suggested an association between the presence of the T-allele at position Taq-1 and the occurrence of AP. The allele T appeared almost three times more often in the AP group (OR = 2.61; 95% CI: 1.68–4.03; *p* < 0.0001) than in the alcohol-abuse control group. Also, the presence of the TT genotype was four times more frequent in the AP group (OR = 4.55; 95% CI: 1.69–12.20; *p* = 0.003) in comparison to the control group. Detailed data for the AP and control groups are shown in Table 1 and Table 2.

## 3. Discussion

Combined genetic, metabolic, and environmental factors all contribute to the development and re-occurrence of acute and chronic pancreatitis [35]. To the best of our knowledge, this is the first examination and comparison of *VDR* gene polymorphism in patients diagnosed with acute pancreatitis. Our study showed higher levels than the accepted reference points for bilirubin, alanine transaminase (ALT), and aspartate transaminase (AST) in the AP-patients (1.9 mg/dL, 155.7 IU/L, and 155.2 IU/L, respectively). This may be associated with liver damage resulting from AP-group alcohol abuse, as high alcohol consumption/addiction is considered a major cause of AP [4]. AP patients also had increased amylase activity and significantly higher lipase activity that indicate pancreatic dysfunction. Their additional significantly increased C-reactive protein (CRP) levels demonstrate that this process can be enhanced by ongoing inflammation.

The average level of vitamin D in the AP and control groups (44.2 and 47.7 nmol/L, respectively) was similar, as noted in alcohol-abuse patients [12]. Although vitamin D deficiency in people abusing alcohol was noted before, the review of Tardelli et al. [36] suggests that data concerning vitamin D levels in alcohol-use patients are controversial. Ogunsakin et al. [37] found that vitamin D (25(OH)D_3_), and its active form, (1,25(OH)_2_D_3_), were significantly reduced in alcohol-abuse patients. Also, ethanol-fed mice showed reduced levels of (1,25(OH)_2_D_3_). These conflicting data suggest that, in selected cases, the analyzed SNPs may be responsible for the observed deficiency of vitamin D responsiveness [38,39].

Our study complements the analysis of AP being a metabolic disorder with genetic factors. We have focused on *VDR* polymorphism rather than vitamin D concentration because of its potential final effect on glucose metabolism. Forouhi et al. [40] suggested that the direct effect of vitamin D on the secretory function of pancreatic cells is through their *VDR*s, and this is suggested to be the explanation for the association between a lower serum vitamin D status and a high risk of hypoglycemia and insulin resistance. Vitamin D action through its receptors may also be responsible for the regulation of insulin secretion by the β-cells in a glucose-dependent manner [41]. Abnormalities in these processes, therefore, lead to glucose metabolism disorders [42,43]. Unexpectedly, we found an increased glucose concentration to a mean of 127.4 mg/dL in AP-patients compared to the reference values (Table 3). This may be a result of glucose homeostasis dysfunction, leading to subsequent inflammation, which is consistent with the clinical parameters established in our AP group where we detected high CRP levels in the 4.4 mg/dL range (Table 3).

Vitamin D was also suggested as an immune modulator because of the existence of *VDR*s in activated T lymphocytes, macrophages, and thymus tissue [44,45]. Palomer et al. stated that inflammatory factors have often been associated with insulin resistance and β-cell failure [46]. Moreover, vitamin D reduces oxidative stress through the induction of an antioxidant activity by itself [47,48], and, importantly, oxidative damage has been implicated in acute pancreatitis initiation [49,50]. Because *VDR* is strongly expressed in pancreatic beta cells [51], its signaling may be reduced or even activated dependent on the occurrence of specific SNPs in AP similar to what has been described in the progression of some tumors [52,53,54]. Reduced vitamin D receptor signaling might be a potential mechanism underlying increased foam cell formation, resulting in an accelerated development of cardiovascular disease in diabetic subjects [55]. 

Here, we used SNP analysis to identify differences in the frequency of Apa-1, Taq-1, Bsm-1, and Fok-1 genotypes/alleles in the *VDR* gene between AP and alcohol-abuse patients. These SNPs are located near the untranslated region and are possibly linked to a poly-A-microsatellite repeat that could affect *VDR* mRNA stability. The frequency of alleles and the distribution of genotypes in AP and alcohol-abuse controls for Bsm-1 (0.38 for B), Apa-1 (0.56 for A), and Fok-1 (0.59 for F) (Table 1 and Table 2) was comparable with the data in a Caucasian population presented by Uitterlinden et al. [33]. These authors found polymorphisms of 0.66 of F in Fok-1, 0.42 of B in Bsm-1, and 0.44 of A in Apa-1. In our study, only the polymorphism in Taq-1 was increased (0.61 in T) in AP (Table 2) compared to the value of 0.43 for T as described previously [33]. Polymorphisms affecting the vitamin D and *VDR* axis are associated with an ongoing degree of inflammation associated with the release of pro-inflammatory cytokines, possibly resulting from modulation of the inflammasome, alterations of gut permeability, and microbial translocation, as suggested by Al-Daghri et al. [56].

Our results suggest that allele *T* in the Taq-1 polymorphic site of the *VDR* gene is almost three times more frequent (OR = 2.61 (95% CI: 1.68–4.03, *p* < 0.0001) in acute pancreatitis patients than in alcohol-abuse controls. *VDR* polymorphism may thus play an important role in vitamin D metabolism independently of the actual vitamin D plasma concentration. Polymorphisms in Bsm-1, Taq-1, Apa-1, and Fok-1 were associated with renal diseases, cancer, neurolithiasis diabetes, asthma, atopic dermatitis, and autism [33,34,57,58,59,60,61,62,63]. To our best knowledge, there was no research focused on *VDR* polymorphism in acute pancreatitis. Only Fok-1 polymorphism was noted in correlation with pancreas allograft [64].

From our results, it can be inferred that, despite a polymorphism in the *VDR* receptor gene (Taq-1), both AP and the control groups showed no difference in plasma concentrations of vitamin D (Table 3). This implies that the interaction between the active metabolite of vitamin D (1,25-dihydroxyvitamin D) and the receptor is responsible for the final effect. Therefore, polymorphisms in the vitamin D receptor gene may have an impact on the development of acute pancreatitis due to the lack of a protective role of vitamin D.

There are several limitations in our study that we will address in subsequent studies. Our results were obtained in a group of 129 AP patients, and we need to repeat and expand these studies in larger replication analyses. Moreover, the *VDR* is part of the nuclear receptor family of transcription factors. Upon activation by vitamin D, a heterodimer is formed between the *VDR* and the retinoid-X receptor, and this complex interacts with vitamin D responsive elements in the DNA, thereby driving expression or transrepression of selected genes. It would, therefore, be of value to analyze also the retinoid-X receptor (*RXR*) gene for the occurrence of SNPs. However, so far, a polymorphism in the *RXR* gene was only found in hyperlipidemia and type 2 diabetes [65]. In addition, we could not extensively compare vitamin D plasma concentrations in controls and AP groups because of their different lifestyles, including diet, sun exposure, and vitamin D supplementation. However, our earlier research [34] did show a lack of a significant correlation between serum 25-hydrohyvitamin D (25(OH)D_3_) levels and *VDR* polymorphism, probably reflecting that plasma vitamin D levels reflect an inactive form of the functional vitamin D3. Therefore, subsequent work will include polymorphisms in other genes affecting the metabolism of vitamin D3.

The prevalence of acute pancreatitis is still increasing and patients are insufficiently diagnosed, necessitating the development of new analytical methods. Here, we added new insights into the putative role of SNPs in *VDR* in pancreatitis development, and, therefore, this study contributes to individualized research on the interaction and impact of environmental factors, including nutrition, in pancreatic secretory disorders. 

## 4. Materials and Methods

### 4.1. Ethics and General Study Information 

Our study comprised of 239 individuals (57 females and 182 males) assigned to control (C) or test groups (AP—acute pancreatitis group). Patients were recruited by specialists at the Department of General Surgery and Oncology of the Warmia and Mazury University Hospital in Olsztyn in 2011–2017. Controls were recruited by specialists at the Department for the treatment of alcohol dependent patients, including patients with other dysfunctions, and the Department for the treatment of withdrawal syndromes of the Jozef Babinski Specialist Psychiatric Hospital in Krakow in 2015. Test patients and controls were of Polish ethnic origin. All patients were treated according to the Patient Right Protection Act of our institution and international guidelines, and the Local Bioethics Committee approved our study (No 13/2016, 27 April 2016).

### 4.2. Controls and AP Group Characteristic

The test group included 129 patients (38 females and 91 males) with diagnosed AP (mean age ranging from 28 to 76 years; average 52.4). These patients presented for treatment 8–36 h after the onset of associated pain and vomiting or emetic reflex. Alcohol abuse was the etiological factor in all AP patients. Exclusions from the study included patients with chronic circulatory system, liver, kidney, and lung disease. Blood samples were taken from the forearm vein for the panel of biochemical tests evaluating pancreatic enzymes, and additional tests assessed general health condition and performance of individual systems and organs.

Laboratory tests were performed upon arrival at the hospital and at 48 h after admission. A computed tomography (CT) scan was performed on all patients within 48 h after arrival at the hospital for detection of the development of fluid collections, the extent of inflammation, and necrotic changes. Acute Physiology and Chronic Health Evaluation (APACHE) II scores were calculated using data from the first 24 h after admission. Serum CRP levels were measured at admission. 

The general health of included patients and the course of their AP disease was assessed by evaluating 4 to 16 points on the APACHE II scale. Abdominal CT with contrast (intravenous and per os) evaluated morphological changes in the pancreas and surrounding environment. Patient evaluation via imaging scales predicting acute pancreatitis severity and the development of pancreatic complications was performed 3–4 days after the onset of symptoms, then again after 10–12 days treatment. Microbiological analyses of stool samples of both the control and AP group did not reveal any significant difference. Possible small remaining fluid reservoirs prior to surgical intervention and puncture and self-absorption mechanisms were monitored by ultrasound. The control group comprised of 110 individuals (19 females and 91 males), with a mean age ranging from 23 to 58 years (average 44.2). All patients met the DSM IV criteria for alcohol dependence. All participants (AP and Control group) in this study were Caucasians. The characteristics of AP patients and controls and laboratory parameters established during hospitalization are shown in Table 3.

### 4.3. Polymorphism of VDR Genes in Control and AP Groups 

DNA was isolated from peripheral blood using a GeneJET™ Whole Blood Genomic DNA Purification Mini Kit (Thermo Scientific, Waltham, MA, USA) according to the manufacturer’s instructions. Bsm-1, Taq-1, Apa-1, and Fok-1 *VDR* polymorphisms were assessed by polymerase chain reaction-restriction fragment length polymorphism (PCR-RFLP). Primers examining the polymorphism in Fok-1 were as previously described [34,66] with slight modifications [67], while primers for Bsm-1, Taq-1, and Apa-1 were designed with the Primer3 application (http://bioinfo.ut.ee/primer3-0.4.0/). The primer specificity was verified with the BLAST algorithm, and primer sequences used for amplification of Fok-1, Bsm-1, Taq-1, and Apa-1 restriction enzyme polymorphisms are listed in Table 4.

PCR amplification was conducted in a thermal cycler according to the following program: Initial denaturation: 94 °C for 3 min, proper denaturation: 94 °C for 30 s, attaching the starters at 61 °C for all genes for 30 s, synthesis: 72 °C for 30 s, final synthesis: 72 °C for 5 min, number of cycles: 40, cooling: 4 °C. The mixture in the volume of 25 μL consisted of DreamTaq™ Green Master Mix (Thermo Scientific), specific primers, the DNA matrix, and ultrapure water (Sigma-Aldrich, Saint Louis, MO, USA). The yield and specificity of PCR products were evaluated by electrophoresis in 1.5% agarose gel (Promega, Fitchburg, MA, USA) and staining with GelGreen Nucleic Acid Gel Stain (Biotium, Hayward, CA, USA).

Amplified fragments were digested with the appropriate restriction enzyme (Thermo Scientific) according to the manufacturer’s instructions, and visualized on a 2.5% agarose gel. DNA sequencing of random chosen samples after amplification was used to confirm proper genotyping.

### 4.4. Statistical Analysis

The mean values in the control and AP groups were compared using a student’s *t*-test. The frequency distribution of common risk factors for AP are presented as the mean ± SD. The genotype distribution among subjects was analyzed for Hardy-Weinberg equilibrium (HWE) using the chi-square test, and genotype and SNP allele frequencies were compared in AP patients and control groups by a Fisher’s test. Odds ratios (ORs) and 95% confidence intervals (CIs) were calculated using logistic regression analysis, and used to compare both allele frequencies in alcohol-abuse controls and AP patients, and allele frequencies between females and males. The risk of AP development was estimated via the wild-type genotype vs. the wild/mutant and mutant-type genotypes. Only all 4-genotyped SNPs probes were used in the calculations. Statistical analysis was conducted on GraphPad Prism software (v 6.01; San Diego, CA, USA), with ≤0.01 *p*-value considered statistically significant.

## Figures and Tables

**Table 1:** Genotype frequencies of Apa-1, Bsm-1, Fok-1, and Taq-1 in *VDR* gene polymorphisms in the studied groups and the association with AP.

Genotype	AP *n* (%)	Control *n* (%)	OR (95% CI) AP vs. Control	*p*-Value
Apa-1 (rs7975232)				
AA (TT)	51 (39.5)	30 (27)	1.0	
Aa (TG)	51 (39.5)	53 (48)	1.76 (0.98–3.19)	0.06
aa (GG)	27 (21)	27 (25)	1.70 (0.84–3.41)	0.14
AA vs. Aa + aa			1.73 (1.14–2.63)	0.009
Bsm-1 (rs1544410)				
BB (GG)	12 (9)	16 (15)	1.0	
Bb (GA)	66 (51)	58 (53)	0.66 (0.29–1.51)	0.32
bb (AA)	51 (40)	36 (33)	0.53 (0.22–1.25)	0.15
Bb + bb vs. BB			1.72 (0.97–3.07)	0.06
Fok-1 (rs2228570)FF (CC)	51 (40)	39 (35)	1.0	
Ff (CT)	57 (44)	47 (43)	1.07 (0.61–1.90)	0.79
ff (TT)	21 (16)	24 (22)	1.49 (0.73–3.07)	0.27
FF vs. Ff + ff			1.45(0.90–2.34)	0.12
Taq-1 (rs731236)TT (TT)	56 (43)	27 (25)	1.0	
Tt (TC)	64 (50)	63 (57)	2.05 (1.11–3.77)	0.02
tt (CC)	9 (7)	20 (18)	4.55 (1.69–12.20)	0.003
TT vs. Tt + tt			2.61 (1.68–4.03)	<0.0001

Genotype frequencies of *VDR* SNPs were determined in the control group, alcohol group, and patients with AP, and associations with AP.

**Table 2:** *VDR* SNPs frequency of alleles in the control group and patients with AP.

*VDR* Polymorphism	Allele	AP *n* = 129	Control *n* = 110	Study Population (AP + Control) *n* = 239
Apa-1 (rs7975232)	A (T)	0.59	0.51	0.56
	a (G)	0.41	0.49	0.44
Bsm-1 (rs1544410)	B (G)	0.35	0.41	0.38
	b (A)	0.65	0.59	0.62
Fok-1 (rs2228570)	F (C)	0.62	0.57	0.59
	f (T)	0.38	0.43	0.41
Taq-1 (rs731236)	T (T)	0.68	0.53	0.61
	t (C)	0.32	0.47	0.39

**Table 3:** Distribution of selected characteristics in acute pancreatitis patients and alcohol-abuse control group.

Characteristic	Alcohol	AP Group	*p*-Value
	*n* = 110	*n* = 129	
Age (years)	44.2 (±8.0)	52.4 (±10.3)	0.09
Body mass (Kg)	71.3 (±8.6)	75.6 (±8.7)	0.73
Amylase activity in serum (IU/L)	114.4 (±61.1)	1647.5 (±636.5)	<0.001
Lipase (IU/L)	131.1 (±45.8)	1446.7 (±814.6)	<0.001
Bilirubin (mg/dL)	0.9 (±0.7)	1.9 (±0.87)	<0.001
Glucose (mg/dL)	85.5 (±12.1)	127.4 (±33.9)	<0.001
AST (IU/L)	70.2 (±51.7)	155.2 (±71.6)	<0.001
ALT (IU/L)	55.2 (±9.7)	155.7 (±30.2)	<0.001
CRP (mg/dL)	0.58 (±1.0)	4.4 (±1.4)	<0.001
25-hydroxyvitamin D (nmol/L)	44.2 (±17.1)	46.7 (±18.4)	0.35
Female (%)	17.0	29.0	
APACHE II scale	n.a.	4.4 (±1.4)	

Values are as expressed as mean ± SD; n.a.: not applicable.

**Table 4:** Primers for *VDR* SNPs and polymerase chain reaction-restriction fragment length polymorphism (PCR-RFLP) conditions.

SNP	Primer Sequence	Restriction Enzyme	PCR/RFLP Products (bp)
Apa-1	TaqF: 5′-ggatcctaaatgcacggaga-3′ TaqR: 5′-aggaaaggggttaggttgga-3′	FastDigest Apa-1	aa: 484, 146 AA: 630 Aa: 630, 484, 146 PCR product: 630
Bsm-1	BsmF: 5′-cggggagtatgaaggacaaa-3′ BsmR: 5′-ccatctctcaggctccaaag-3′	FastDigest Bsm-1	bb: 243, 105 BB: 348 Bb: 348, 243, 105 PCR product: 348
Fok-1	FokR: 5′-atggaaacaccttgcttcttctccctc-3′ FokF: 5′-agctggccctggcactgactctggctct-3′	FastDigest Fok-1	ff: 198, 69 FF: 267 Ff: 267, 198, 69 PCR product: 267
Taq-1	ATaq1F: 5′-ggatcctaaatgcacggaga-3′ ATaq1R: 5′-aggaaaggggttaggttgga-3′	FastDigest Taq-1	tt: 225, 200, 205 TT: 425, 205 Tt: 425, 225, 200, 205 PCR product: 630
